# Linking household surveys and facility assessments: a comparison of geospatial methods using nationally representative data from Malawi

**DOI:** 10.1186/s12963-020-00242-z

**Published:** 2020-12-10

**Authors:** Michael A. Peters, Diwakar Mohan, Patrick Naphini, Emily Carter, Melissa A. Marx

**Affiliations:** 1 Johns Hopkins Bloomberg School of Public Health, 615 N. Wolfe Street, Baltimore, MD 21205 USA; 2 Malawi Ministry of Health is the institution, Lilongwe, Malawi

**Keywords:** Spatial linkage, Linking, Small area estimation, DHS, Misclassification, Malawi, GIS, Family planning

## Abstract

**Background:**

Linking facility and household surveys through geographic methods is a popular technique to draw conclusions about the relationship between health services and population health outcomes at local levels. These methods are useful tools for measuring effective coverage and tracking progress towards Universal Health Coverage, but are understudied. This paper compares the appropriateness of several geospatial methods used for linking individuals (within displaced survey cluster locations) to their source of family planning (at undisplaced health facilities) at a national level.

**Methods:**

In Malawi, geographic methods linked a population health survey, rural clusters from the Woman’s Questionnaire of the 2015 Malawi Demographic and Health Survey (MDHS 2015), to Malawi’s national health facility census to understand the service environment where women receive family planning services. Individuals from MDHS 2015 clusters were linked to health facilities through four geographic methods: (i) closest facility, (ii) buffer (5 km), (iii) administrative boundary, and (iv) a newly described theoretical catchment area method. Results were compared across metrics to assess the number of unlinked clusters (data lost), the number of linkages per cluster (precision of linkage), and the number of women linked to their last source of modern contraceptive (appropriateness of linkage).

**Results:**

The closest facility and administrative boundary methods linked every cluster to at least one facility, while the 5-km buffer method left 288 clusters (35.3%) unlinked. The theoretical catchment area method linked all but one cluster to at least one facility (99.9% linked). Closest facility, 5-km buffer, administrative boundary, and catchment methods linked clusters to 1.0, 1.4, 21.1, and 3.3 facilities on average, respectively. Overall, the closest facility, 5-km buffer, administrative boundary, and catchment methods appropriately linked 64.8%, 51.9%, 97.5%, and 88.9% of women to their last source of modern contraceptive, respectively.

**Conclusions:**

Of the methods studied, the theoretical catchment area linking method loses a marginal amount of population data, links clusters to a relatively low number of facilities, and maintains a high level of appropriate linkages. This linking method is demonstrated at scale and can be used to link individuals to qualities of their service environments and better understand the pathways through which interventions impact health.

## Background

Facility assessments and household surveys are two commonly employed study designs and can provide rich information about how interventions result in health impacts when combined. Facility-based surveys such as the Service Provision Assessment (SPA) are useful at the health system level for measuring inputs and structure, processes of care, outputs like service utilization, and some health outcomes at the health facility level (The DHS Program, “SPA Overview” [1]). Household surveys like the Demographic Health Surveys (DHS) or the Multiple Indicator Cluster Surveys (MICS) are important for obtaining estimates of population-based health outcome (e.g., intervention coverage) and impact (e.g., morbidity and mortality) measures (The DHS Program, “Demographic and Health Surveys” [2]; UNICEF, “Multiple Indicator Cluster Surveys” [3]). Linking inputs and processes with impact at the most granular level possible—i.e., individual health data with health facility/provider characteristics—can support comprehensive evaluation of health system performance [4, 5]. As a result of these linkages, researchers can bring together supply- and demand-side information to conduct health systems research, such as deriving quality-adjusted coverage levels, which contribute to understanding of effective coverage of services and tracking of progress towards Universal Health Coverage [6]. Overall, these linkages can be used to determine the relationship between services delivered by the local health system and relevant population health outcomes.

Due in part to the frequency and methodological rigor of these surveys, there is an increased interest in combining data on health outcomes with relevant data on the health service environment. Linking survey data can be an attractive method to efficiently maximize the use of existing data and improve the strength of analysis [7, 8]. Specifically, granular geographic linkages can reduce the effect of ecological bias that can exist when results are aggregated at higher levels, such as districts or states, by focusing on individuals or households and the specific facilities that they are likely to use. Additionally, geospatial linking can serve as a tool for enhancing monitoring and evaluation functions of programs through rigorous disaggregated assessment, localizing successes and failures of interventions, and informing a bottom-up approach to program design, implementation, and mid-course program adjustments.

**Fig. 1:**
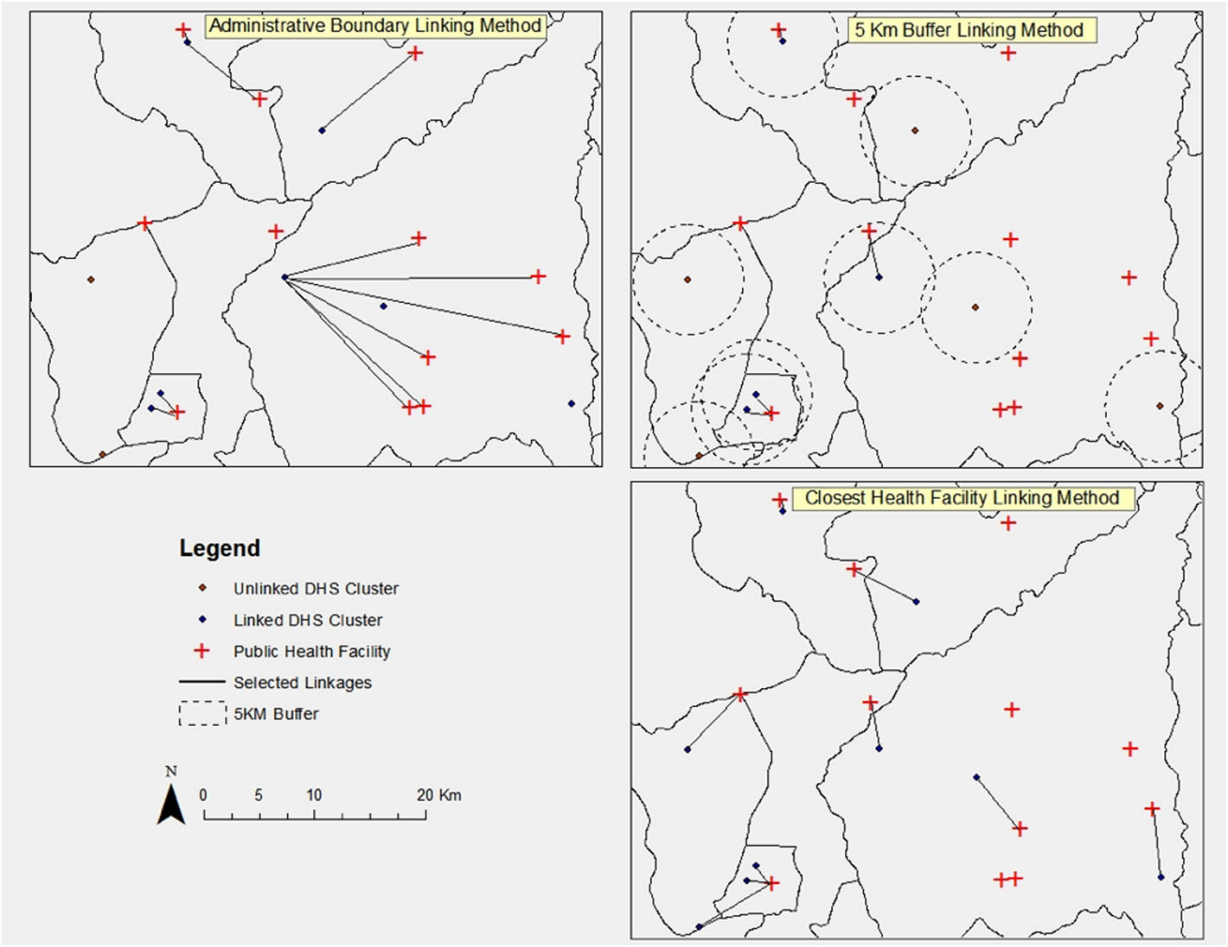
Depictions of common current linking methodologies. The top-left panel demonstrates linkages for selected clusters and all facilities within an administrative district. The top-right demonstrates linkages for clusters and all facilities located within a 5-km buffer. The bottom-right panel demonstrates linkages for clusters and the single closest facility

There is extensive literature detailing the methods and describing the potential for linking facility and household data, including a systematic review [7] and several methods papers published by USAID, ICF Macro, and MEASURE Evaluation [5, 9–11]. The main methods of linking data from household and facility surveys can be categorized into two classes: (i) indirect/ecological linking, where health care-seeking behavior is linked to facilities or providers by resident geographic criteria, and (ii) direct linking, where individuals are linked with the exact provider or facility where they sought care [7]. The direct linking method is the most precise method for linking and enables researchers to have reasonable certainty that households are linked with the provider from which they received care. However, indirect linking is usually most feasible for large scale surveys and is most frequently used because information on specific facility use is not collected in periodic household surveys like the DHS and MICS [7]. Although at least eight techniques for indirect linking of population and facility surveys have been described [9], the three most frequently applied methods for indirect linking have included linking populations to the closest facility (Fig. 1, bottom-right panel ), linking populations to all facilities within a geographic distance (Fig. 1, top-right panel ), and linking populations to all facilities within a certain geographic area (Fig. 1, top-left panel ) [7].

These methods have been included in a battery of sensitivity analyses to demonstrate methodological considerations in the context of linking households to family planning services in Rwanda [12]. The extent to which these methods are effective can be assessed by comparing the last reported source of an individual’s family planning method with the types of health facility that an individual is geographically linked with through the various methods. Results from three such comparisons in Rwanda found that the administrative boundary linking method most frequently linked the highest percent of women to their last reported source of family planning, followed by the 5-km buffer method, with the closest facility method performing the poorest [12]. Across methods, linking success varied by source of last modern^1^ contraception: across methods, over 90% of women who last received modern contraception from a health center were linked to a health center, while hospital users and dispensary users were linked to their last source of modern contraception much less frequently [12].

These results demonstrate some of the strengths and weaknesses associated with each of these popularly used approaches. While the closest facility linkage method may be useful depending on the type of service under study (i.e., emergency services), it limits the service environment to a single facility and was deemed less effective for linking households to their last source of family planning in a similar context in Sub-Saharan Africa [12]. The buffer linking method linked a greater proportion of women to their last reported source of family planning than the closest facility linkage but demonstrated limited success when household locations were geographically displaced—intentionally “shifted” within predefined parameters to preserve confidentiality—especially when the range of displacement is equal to the radius of the buffer [12]. The administrative boundary method is a blunt tool that is effective in linking a household cluster to a broad service environment, especially since many displacement methods attempt to keep clusters within their true administrative boundary [13]. However, ecological bias is high when attempting to analyze outcomes and exposures at the administrative level, as each cluster is linked to every single facility within the same administrative unit, creating a homogenous service environment within an administrative boundary. Furthermore, this method assumes that individuals do not cross administrative boundaries when seeking care, which may reflect reality in some contexts, for example facility restrictions based on insurance coverage, but not others.

While strengths and limitations of indirect linking methods have been demonstrated in a relatively limited sample (the Skiles study had a sample of 767 women from 185 clusters), there is a need to repeat this analysis with a larger sample size and at a national scale in other settings. The limitations of existing methods also highlight a need for new geospatial approaches to form appropriate, localized indirect linkages. This paper compares the performance of some common linking methods at a national level in Malawi in addition to a previously undescribed method for linking by theoretical catchment area. Findings from this analysis should be used to inform analyses that link individuals or households from displaced survey cluster locations with undisplaced health facility locations and where information about health facility type is available. As more household surveys release data with displaced cluster coordinates and facility censuses become widely available, this scenario is likely to become increasingly relevant over time.

### Malawi context

Malawi is a relatively small, mostly rural, landlocked country in southeast Africa with a burgeoning population that has grown from 4 million people in 1966 to over 16.4 million people today [14]. The government of Malawi is committed to decelerating Malawi’s population growth and improving the health of the population by expanding the use of family planning services [15]. The country is administratively divided into 28 districts, and its public hospitals and health centers all provide family planning services. Public health facilities are generally located following the population distribution of the country, clustering in the four main urban areas: Lilongwe, Blantyre, Mzuzu, and Zomba. Outreach workers (health surveillance assistants and community-based distribution agents) and village clinics and health posts (lower level facilities that support nearby health clinics and hospitals) supplement these static health facilities and provide family planning in more rural settings. No major topographical features restrict movement (except for access to the small island district of Likoma), and there is a major highway running north to south down the spine of the country. The government would like to be able to describe formal sector service environments and link individual health data to facility-level characteristics, especially in the context of family planning services to determine if family planning programs are having desired impacts.

## Methodology

### Data sources

#### Service environment data

Between May 2015 and October 2016, a census, or master list, of all health facilities was collected through an initiative of UNICEF and the Malawi Ministry of Health (MOH). The master list contains all health facilities and hospitals providing free services in Malawi, in addition to their related outposts and village clinics. The master list provides information for all public government services, the Christian Health Association of Malawi (CHAM), which is publicly funded but administered by religious non-governmental organizations (NGOs), and some other faith-based and private facilities. These facilities and their outreach locations generally provide family planning services, although some facilities have a policy of not providing these services based on religious beliefs. The master list dataset includes location information (i.e., latitude and longitude in decimal degrees collected by GPS in WGS 1984 datum) of all hospitals, health centers, health posts, village clinics, and other lower-level services associated with either a hospital or health center, as well as the organization that operates the facility. While the location of hospitals and health centers is not likely to change, it is important to note that the location of these village clinics and health outposts can be shifted upon local leader agreement in order to better meet demands of the community.

#### Population data

Results from the 2018 Malawi Population and Housing Census were not published as of the time of analysis, so the 2008 census is the most recent national population estimate in Malawi. The census divides Malawi into four administrative levels: national boundaries, district-level boundaries, traditional authority/sub-chieftain (TA) boundaries, and enumeration area (EA) boundaries (NSO 2008). The 2015 Malawi Demographic Health Survey (MDHS) is the fifth survey in the Malawi DHS series and provides population-level health estimates, including data useful in monitoring and evaluating population, health, and nutrition programs. The MDHS 2015 used a sample frame from enumeration areas in the 2008 census, and a two-stage sample selection process to randomly select and interview a total of 26,361 households, including 24,562 female respondents, from 850 enumeration areas to provide estimates of key health indicators that are representative both nationally and for each of Malawi’s 28 districts [16].

DHS clusters are the smallest unit of data collection for which geographic location information are available. At the time of data collection, coordinates are collected from the central location of the selected enumeration area, condensing the enumeration area polygon into a single point location, called a cluster. Each cluster represents 100 to 300 households, of which 20–30 households are randomly selected for survey participation [13]. Health estimates obtained at these clusters are a random sample of the entire enumeration area and are generally representative of population health at the level of the enumeration area [17]. To protect the privacy of survey participants, the locations of the clusters are purposefully randomly displaced [13]. Rural clusters are displaced up to 5-km away in a random direction from the original location, and 1% of these rural clusters are randomly selected and displaced up to 10 km away. Urban clusters are displaced randomly up to 2 km from the actual location in a random direction. All displacement is checked manually to ensure that clusters remain within the administrative boundary in which they are actually located, at the district level for the Malawi context [13].

Rural households comprise 83% of Malawi’s population [14] and are the focus of this analysis. Individuals in urban environments have more private sector options, a higher facility density, and more transportation options than their rural counterparts, making physical proximity a relatively poor predictor of service utilization in urban environments [12]. A large subset of women currently using modern contraception access their contraceptive method through facilities (8440 or 80.44% of modern contraceptive users). The remaining 19.56% of modern contraceptive users receive family planning from a variety of sources, primarily from the NGO, Banja La Mtsogolo (BLM—6.61%), from health surveillance agents (HSAs—4.31%), and from private hospitals and clinics (3.13%—see Additional file 1: Annex 1). This analysis is restricted to women currently using a modern method of contraception who reported a last source of family planning that is eligible for linkage and exist in clusters that are located outside of the geographic administrative boundaries of the Lilongwe and Blantyre city limits (Malawi’s two largest urban areas).

### Approaches for data linkage

All linkages were performed in ArcGIS Desktop (Redland, CA). Four distinct spatial joins linked MDHS 2015 clusters with facilities. Specifically, clusters were linked (i) to the single closest facility, (ii) with any facilities that lie within a 5-km radius of the cluster, (iii) with all facilities within the same district, and (iv) with any facilities whose theoretical catchment area falls within a 5-km radius of the cluster. The methodologies for performing these spatial joins are described in detail elsewhere (see Additional file 2: Annex 2).

**Fig. 2:**
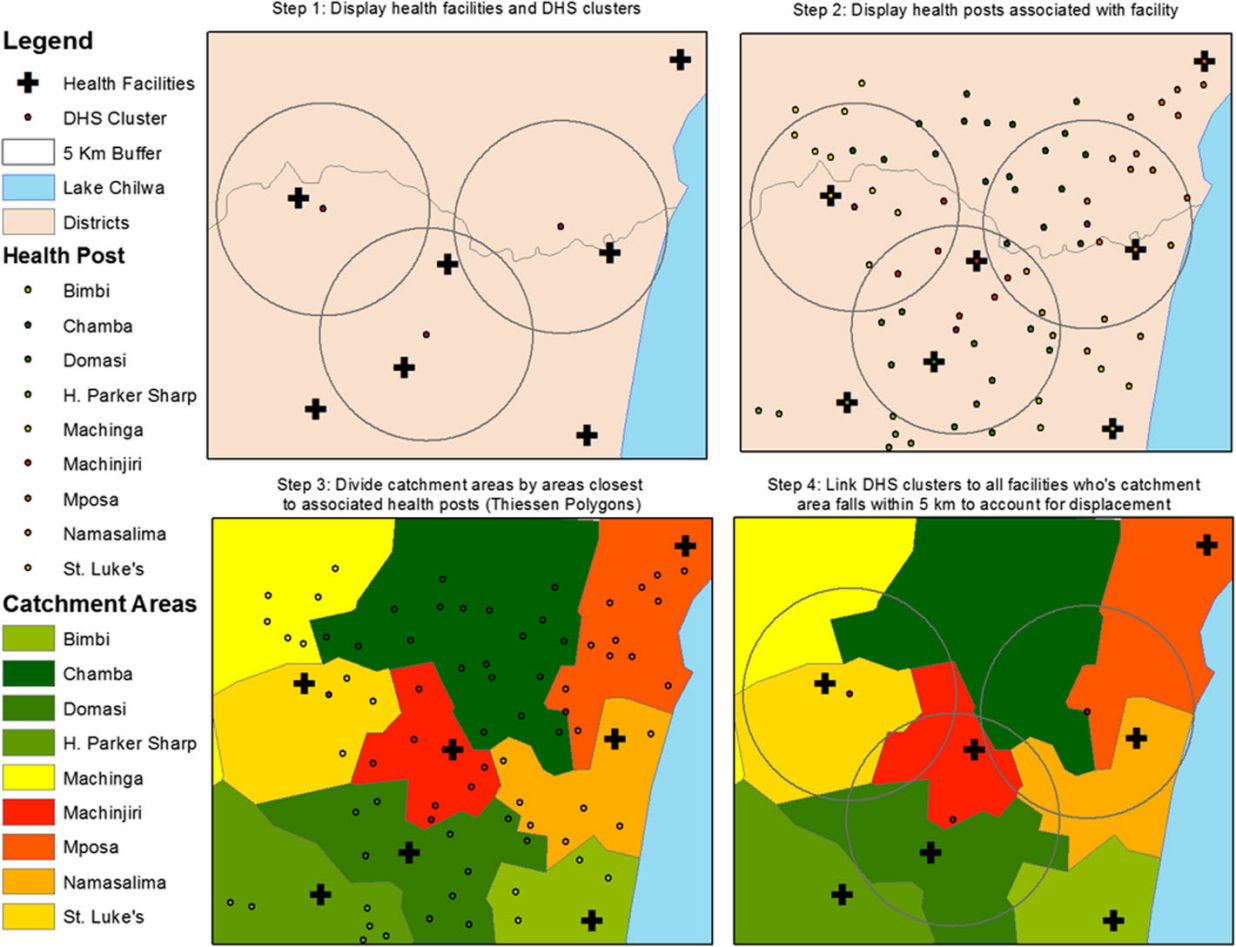
Process for completing a theoretical catchment area linkage. Steps 1–3 demonstrate the process for creating theoretical catchment areas for individual facilities based on Thiessen Polygons created around their lower-level associated health outposts. Step 4 demonstrates the use of 5-km buffers to link clusters to facilities by theoretical catchment area

The fourth technique mentioned was developed for this study to address limitations in existing methods and requires additional description. The “theoretical catchment area linking method” links MDHS 2015 clusters to health facilities based on a theoretical catchment area belonging to a facility, rather than the exact location of a health facility. For research questions requiring an understanding of the population a facility is attempting to serve, a true catchment area method of linking individual and facility data has been described [10]. This method requires the demarcation of a catchment area around a facility to denote that facility’s service area. In the context of Malawi, no official geospatial data exist that outline the true catchment area of a particular health facility. Instead, a theoretical catchment area containing all of a facility’s associated lower-level outreach locations, village clinics, and outreach posts can be created around a particular health center or hospital. These catchment areas should not overlap and represent the boundaries of a facility’s service area, meaning that households existing within the boundaries of the catchment area should ideally be serviced by the facility. Taken together, in Malawi, these theoretical catchment areas represent a visual representation of the public health system (including CHAM-operated facilities) and can be a useful planning tool in their own right. Theoretical catchment areas can be created in a geographic information system (GIS) for each health facility and hospital in the master facility list by creating Thiessen Polygons around all health facilities and then merging each facility and related, lower-level service locations into spatial units (see Fig. 2 for process; Additional file 2: Annex 2 for instructions).

Ideally, if a household is located within a catchment area, it should be linked with the facility that “owns” that theoretical catchment area. However, due to displacement in the MDHS 2015 clusters, a cluster may not be represented within the catchment area in which it truly is located. To account for this displacement, MDHS 2015 clusters are linked to the facilities that have a catchment area within a 5-km buffer of the cluster (Fig. 2). This buffer is meant to account for most of the displacement in the MDHS 2015 cluster location. Clusters were allowed to link with multiple theoretical catchment areas, and the linkages ignored administrative boundaries. Combining the theoretical catchment area and buffer linking methods ensures that most MDHS 2015 clusters are linked with the cluster in which they would be categorized had displacement not occurred, with the exception of the 1% of clusters which may have been displaced more than 5 km. All four of the linking methodologies were compared following the same processes.

### Comparison of linking methods

Results were compared across the four linking methods based on three metrics of performance: (i) the number of MDHS clusters that remain unlinked to any facilities; (ii) the average, and standard deviation of, number of facilities each cluster was linked to; and (iii) the percent of women (currently using modern contraception) who were linked to a facility of a type that matched their last reported source of family planning. Each of the metrics is included to highlight features of the various linking methodologies and have been previously described in studies that compare geographic linking methods [12]. The first metric indicates the amount of individual-level data that is likely to be lost when performing a linkage, as unlinked data must generally be dropped in analyses that require both population and facility data. The second metric indicates the extent of the service environment that each cluster is linked to. A linking technique is most useful when it links an individual to the single actual facility where that person received care; any additional linkages to facilities would provide unnecessary noise since they were not actually used by that individual. Thus, the number of facilities linked per cluster indicates how localized the performance of various methodologies is but does not indicate whether individuals are linked to facilities from which they actually receive care. The final metric indicates whether the linkage method is performing well in terms of linking people to the type of facility from which they last reported receiving care, a proxy for demonstrating ability to link individuals to the facility from which care was actually received.

The first two metrics are self-explanatory and are derived using summary statistics on the joined tables that are the outputs of the various linking methods in GIS. Assessing the third performance metric requires combining individual level data from the MDHS 2015 with the outputs of the GIS processes. Specifically, the indicator for source of modern contraception from the MDHS 2015 is used, which describes the place where the modern method currently being used was obtained the last time it was acquired. There were 24 sources of modern contraception identified by participants, of which 5 can be linked to sources of contraception that were included in the Malawi master list of facilities: government hospitals, government health centers, government health posts, CHAM hospitals, and CHAM health centers. The majority of women using modern contraceptive methods (80.44%) listed one of these 5 sources as their last source of family planning in MDHS 2015 (see Additional file 1: Annex 1). Of the sources that are excluded in this study, some are provided by governments but lack a distinct, static location (e.g., HSAs and community-based distribution agents who may deliver community-based services), other formal, non-government providers (e.g., private hospitals/clinics or major NGOs like BLM), or informal sources (e.g., friends/relatives or shops). A woman was considered appropriately linked if her last reported source of modern contraceptive method matched the type of a facility to which her cluster was linked. For example, if a woman who responded that she last received modern contraception from a “government health center” is linked to a health center operated by the Ministry of Health in the master facility list, we consider her to have been appropriately linked. Although it is not guaranteed that the woman received contraceptive from this particular health center, this estimate provides an upper bound on the likelihood that a woman was linked to a facility she used for family planning [12]. Due to confusion over the classification of CHAM facilities as hospitals or health centers, all CHAM facilities are treated as one type of source. The number of appropriate linkages is summed and divided by the total number of eligible women to determine the percent of appropriate linkages for each method and across each source type.

## Results

The subset of 817 rural clusters from the MDHS 2015 includes 10,492 women who reported using a modern method of contraception (44.75% of respondents). Of these modern contraceptive users, 8440 women reported last receiving contraceptives from government hospitals, government health centers, government health posts, and CHAM-hospital/health centers and are therefore eligible for the study. The contraceptive mix was similar between the women included and those ineligible for the study, with injections being the primary choice, followed by female sterilization, and implants, together comprising over three quarters of all methods used (see Table 1). The facility master list includes 85 hospitals, 542 health centers, and 8870 health posts/village clinics. Of these, 450 hospitals and health facilities are controlled by the government, 145 are CHAM facilities, and the remaining are NGO-operated.

**Table 1:** Contraceptive mix for rural women included and ineligible for linking exercise

Contraceptive method	Ineligible for study		Included in study	
Injection	805	39.2%	4392	52.0%
Female sterilization	429	20.9%	1388	16.4%
Implants/Norplant	355	17.3%	1857	22.0%
Male condom	289	14.1%	353	4.2%
Pill	101	4.9%	286	3.4%
IUD	36	1.8%	138	1.6%
Lactation	16	0.8%	0	0.0%
Male sterilization	8	0.4%	12	0.1%
Standard days method	7	0.3%	8	0.1%
Female condom	3	0.1%	4	0.0%
Emergency contraceptive	2	0.1%	0	0.0%
Other modern method	1	0.0%	2	0.0%
Total	2052	100.0%	8440	100.0%

Two linking methods, linking to closest facility and to all facilities within the administrative boundary, linked all 817 clusters to at least one facility. The theoretical catchment area method linked all but one cluster to at least one facility. The 5-km method left 288 MDHS clusters unmatched to any facility. In terms of average numbers of facilities linked per cluster, the closest facility linkage predictably linked exactly one facility to each cluster. The administrative boundary method linked clusters with an average of 21.1 facilities, with a wide variation in the number of linkages (standard deviation = 9.53). The 5-km buffer method linked clusters to a mean of 1.4 facilities each with some variation (standard deviation = .75). The theoretical catchment area method on average linked clusters to 3.3 facilities with relatively little variation (standard deviation = 1.55) (see Table 2).

**Table 2:** Overall results of cluster-facility linkages by method

Result	Linkage method			
	Closest facility	5 km	Administrative boundary	Catchment
Number of clusters linked to at least one facility (percent linked)	817 (100.0%)	529 (64.7%)	817 (100.0%)	816 (99.9%)
Average health facilities linked per cluster (SD)	1.0 (0.00)	1.4 (0.75)	21.1 (9.53)	3.3 (1.55)

All methods appropriately linked individuals who used health centers as their last source of family planning more frequently than those who reported hospitals as their last source of family planning. Linking to all facilities within an administrative boundary had the highest overall frequency of appropriate linkages. The theoretical catchment area linked a higher overall percentage of women to their last reported source of modern contraception than the closest facility and the 5-km buffer methods (see Table 3).

**Table 3:** Percent of modern contraceptive users from the Malawi Demographic Health Survey 2015 clusters linked to last source of modern contraception by linkage method

	Closest facility	5-km buffer	Administrative boundary	Theoretical catchment area	Number of women available for linkage
Hospitals	51.6%	47.9%	90.7%	72.9%	2287
Health center	70.6%	54.1%	100%	96.2%	4948
CHAM	60.6%	62.3%	99.7%	82.4%	653
Health posts	72.6%	36.4%	100%	97.6%	552
**Overall**	64.8%	51.9%	97.5%	88.9%	8440

## Discussion

We found that of all methods linking to all facilities in an administrative boundary area is the most effective at maximizing the percent of linkages to the facility where women last received family planning. However, these districts are very large, some covering over 10,000 km^2^. Through this method, women were linked to 21.1 facilities on average, indicating a low level of precision as women were linked to many facilities where they were unlikely to receive care. The closest and 5-km buffer methods had low numbers of average linkages (1 and 1.4 respectively), but also relatively low levels of overall appropriate linkages (64.8% and 51.9% respectively), indicating that these methods may be too limited to capture the facility from which they actually received care, especially after displacement of DHS cluster location. The theoretical catchment area method bridges this gap, appropriately linking 88.9% of all women to their last source of family planning, while linking women on average to 3.1 facilities. Our findings indicate that the theoretical catchment area linking methodology maximizes the amount of data available, does not bluntly match to a large number of facilities, and retains a comparably high level of appropriate linkages.

This study employed four methods for linking women to their last source of family planning based on GIS methodologies. Although three of these methodologies have been performed routinely at regional and more local levels, this is the first time that they have been applied at a national scale with such a large sample. Furthermore, this study demonstrates a new methodology that successfully links women to their last source of modern contraception more precisely than existing methods while maximizing the amount of usable data.

**Fig. 3:**
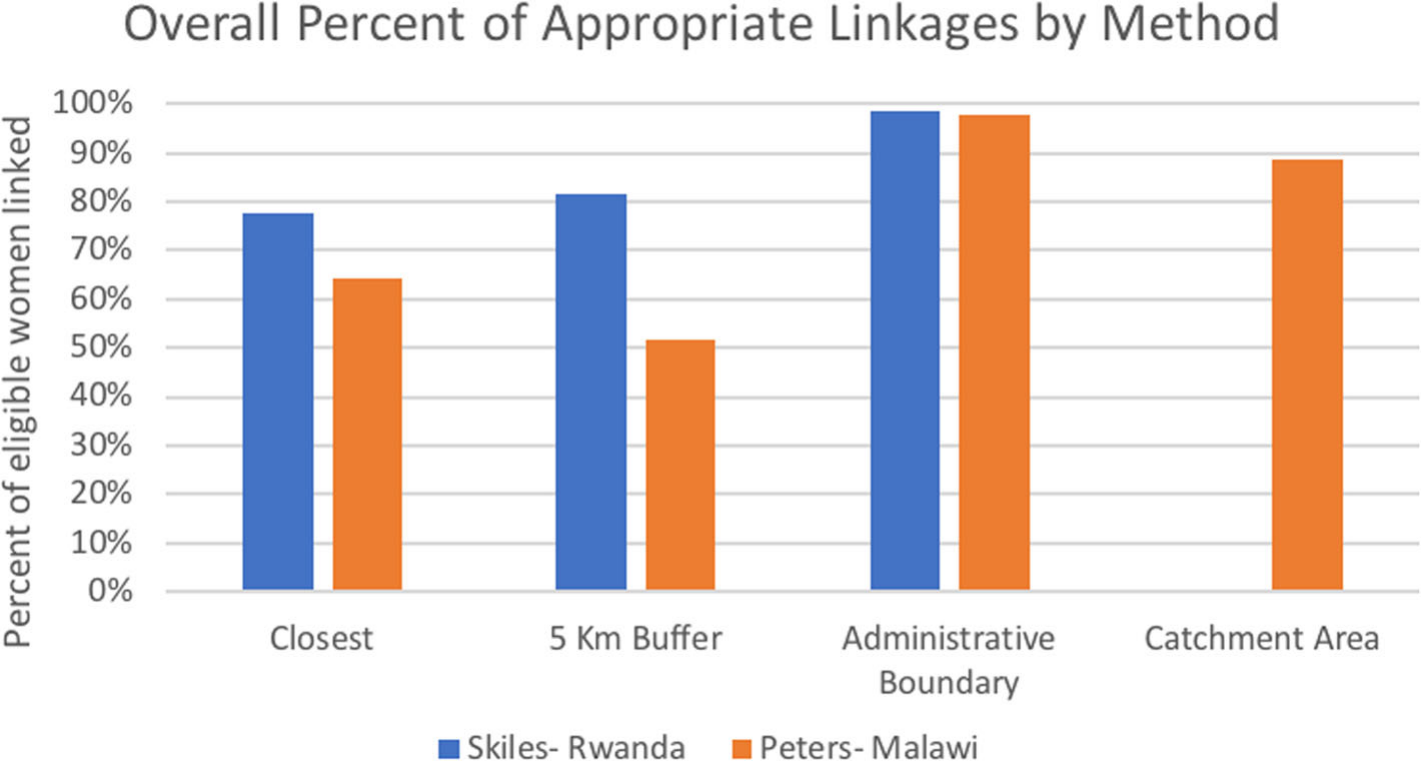
Comparison of linking methods between Rwanda and Malawi: percent of women appropriately linked with a facility matching their last reported source of modern contraceptive

Comparing our findings to Skiles’ findings in Rwanda provides insights about the methods as they are applied to multiple contexts (Fig. 3). The closest facility and 5-km buffer methods performed worse overall in Malawi than in Rwanda. This could have been because our study was employed at a national level, whereas the Rwanda study was confined to a smaller scale, which could have meant that linkages relying on short distances were more appropriate and effective. There also might have been differences in the accessibility of services between rural women in Malawi and Rwanda. In the Skiles study, 90.8% of DHS clusters were within 5 km of any health facility, whereas only 64.7% of clusters were within 5 km of any health facility in the Malawi study. If there are fewer services located close to DHS clusters, linkages based on proximity may be less likely to be appropriate, which was demonstrated through this comparison.

Not all sources of modern contraception identified by MDHS respondents can be linked through the various methodologies. Locations of sources such as private hospitals, mobile clinics, and shops were not available; therefore, they were excluded from analysis, which limits the generalizability of this study, as populations receiving family planning through these sources are likely different than the ones included in our study. Bias also could be introduced if the contraceptive mix was significantly different between the women included in the study and those ineligible for the study, as linking appropriateness may vary based on method used. A separate critique of the technique used to determine if linkages are appropriate is that even if women are successfully linked to the same type of facility (i.e., government health center) where they receive modern contraception, we cannot know for certain that this is the actual facility from which they received modern contraception. In the theoretical catchment method, most people will be linked to at least one public health center due to the relatively dense national distribution of facilities; however, the appropriateness of linkages can be examined at a finer scale through the accuracy of linkages for CHAM facility users. CHAM facilities are more sparsely distributed across the country and likely provide a more direct measure of linkage accuracy than public health facilities. The higher accuracy of CHAM linkages from the theoretical catchment linkage methodology (82.4%) lends strength to the interpretation that individuals are probably linked with the actual public health facility that they received care from using the theoretical catchment area linking technique. Despite this, the linkages deemed to be appropriate in this study may not reflect where individuals actually received care. Future research should compare results of indirect linkages with gold standard methods of linking individuals to where they report receiving care. The expansion of GPS-enabled smartphones with applications that can better predict where individuals received services could facilitate true validation studies and ultimately increase the use of direct linkages between individuals and where they actually received care [18].

Data availability and data quality influence the ability to accurately link population and facility-based surveys [12]. Creating theoretical catchment areas that accurately represent the service environment requires a census of facilities for the study area. In some contexts, it may be difficult to obtain a detailed census of health facilities with associated geographic information. Fortunately, detailed facility censuses are becoming more available in low resource settings as a part of the push for better health informatics [19]. MEASURE Evaluation keeps a periodically updated list of master facility lists and found that 24 low- and middle-income countries (LMICs) currently have a master facility list [20]. RHINO Vision has also compiled a spatial database of health facilities managed by the public health sector in 50 countries in sub-Saharan Africa [19]. A consideration for future linking activities is that many surveys, including the DHS, displace the actual location of surveyed populations to ensure anonymity. While data availability and quality can still be a major hurdle, when available and when appropriate for the research question, the authors recommend using the catchment facility method.

Other notable limitations exist, such as the fact that indirect linking methodologies in general are most effective in analysis involving rural populations. Urban areas generally have more health service options, including private sector options, and transportation options that allow an individual to access health services not close to their home [9] complicating indirect linkages with facilities based on distance. In this study, the inclusion of semi-urban clusters (like Mzuzu and Zomba) could have blurred the divide between rural and urban settings. Appropriate indirect linking methodologies are needed that are tailored for urban area contexts.

Another limitation of this study is that it relied on self-report of where women received family planning services. MDHS 2015 respondents may have difficulty recalling where they received family planning services (e.g., hospital vs health center) or the classification of facilities may be changed over time, which would negatively impact the accuracy of linkages. Furthermore, MDHS 2015 only asks about the last source of family planning for women who are currently using contraceptives. This limits the results to only active users and may introduce bias if there are systematic differences related to the source of contraception for current users compared to women who are not currently using contraception. Despite the fact that the MDHS 2015 and the master facility list were both collected between 2015 and 2016, new facilities may have opened or closed between data collection periods. Additionally, between 2008 and 2018, district boundaries could have changed. Only the administrative boundary linking methodology would have been affected by this change, since other methods ignore district boundaries; thus, we think that our results would largely remain constant.

Indirect linking methodologies can also be further improved through the use of additional data. Incorporating data about the availability of contraceptive methods in the facilities can help make linkages more appropriate. For example, if sterilization is only available at the hospital-level, then extraneous linkages to health centers or health posts can be ruled out as the actual last source of family planning. This use of additional data has been demonstrated to increase the probability of an appropriate linkage in a similar study on injectables in Malawi [21]. In studies where the exact location of clusters is available, clusters may be linked to the single closest health post or theoretical catchment area rather than all theoretical catchment areas within 5 km, increasing the precision of linkages made by this method. Furthermore, when facility-level data such as service availability is available, methods such as Kernel Density Estimation (KDE) can be used to enhance the catchment area technique by creating a more nuanced service environment that better reflects the intensity of services provided [21, 22]. The intensity of family planning program implementation is available at the facility level in Malawi, and while beyond the scope of this linking study, KDE will be utilized in a future study that compares the implementation strength of programming and related health outcomes in Malawi. Additional analyses and studies linking health needs, utilization, and service quality have the potential to improve understanding of health systems phenomena such as achieving effective coverage of health services at a local level [23–25]. Such analyses will be important to better measure progress towards achieving universal health coverage of essential services. We hope that this paper will contribute to improved methods for studying implementation and evaluation for public health interventions and generating evidence for life-saving interventions across multiple socioecological levels.

## Conclusion

This study compares different indirect techniques for linking individuals with service providers at a national scale in a rural LMIC setting. It employs and demonstrates the utility of a new methodology that can be considered alongside established methods. We illustrate here that the theoretical catchment area methodology appropriately links women to their last source of family planning in a high proportion of cases, and includes the vast majority of the clusters in analysis, without linking to every facility in a broad region, thereby addressing some key weaknesses in previous techniques. The ease of these methods, combined with increased availability of geospatial data and computing and programming capacity, can improve the ability of analysis to target communities at a more granular level. The resulting granular level data can inform program planning and mid-course correction and improve how programs respond to the needs and experiences of local communities. Application of this methodology can result in the development of more robust methods of evaluation, providing insights that can ultimately improve the effectiveness of health programs in LMICs globally.

## Supplementary Information

**Additional file 1:.** Annex 1: Eligibility for Comparison Exercise Based on DHS Last Source of Family Planning. Legend: This table presents the number and percent of modern contraceptive users that are eligible for the study and those that are not, by last source of family planning method. It also demonstrates how a linkage was considered to be “appropriate”, when applicable.

**Additional file 2:.** Annex 2: GIS Methodologies. Legend: This document provides guidance on how to conduct the various linkages within a Geographic Information System. The authors hope that this will facilitate reproducibility and expand the use of these methods across settings.

## Data Availability

The MDHS 2015 datasets analyzed during the current study are available in the DHS repository, https://dhsprogram.com/data/. The Malawi master health facility census dataset are available from Patrick Naphini on reasonable request.

## Abbreviations

CHAM: Christian Health Association of Malawi
DHS: Demographic and Health Survey
EA: Enumeration area
GPS: Global Positioning System
GIS: Geographic information systems
HSA: Health surveillance agent
KDE: Kernel density estimation
LMIC: Low- or middle-income country
MDHS: Malawi Demographic and Health Survey (2015)
MICS: Multiple Indicator Cluster Survey
MOH: Malawi Ministry of Health
NGO: Non-government organization
NSO: National Statistics Office of Malawi
SPA: Service Provision Assessment
TA: Traditional authority
USAID: United States Agency for International Development
WGS: World Geodetic System
