# Supplementary material for: Linking household surveys and facility assessments: a comparison of geospatial methods using nationally representative data from Malawi
Source: Popul Health Metr. 2020 Dec 10;18:30. doi: 10.1186/s12963-020-00242-z (PMC7731755; doi:10.1186/s12963-020-00242-z)
Supplement: Supplementary file 1 — Additional file 1:. Annex 1: Eligibility for Comparison Exercise Based on DHS Last Source of Family Planning. Legend: This table presents the number and percent of modern contraceptive users that are eligible for the study and those that are not, by last source of family planning method. It also demonstrates how a linkage was considered to be “appropriate”, when applicable. [file 12963_2020_242_MOESM1_ESM.docx]

### Annex 1: Eligibility for Comparison Exercise Based on DHS Last Source of Family Planning

| **Last Source of FP Method** | **Number of Modern Contraceptive Users citing last source** | **Percent of modern contraceptive users** | **Criteria for match** |
| --- | --- | --- | --- |
| Government health center | 4,948 | 47.16% | Linked to health facility operated by MOH |
| Government hospital | 2,287 | 21.80% | Linked to hospital operated by MOH |
| Government health post / outreach | 552 | 5.26% | Linked to hospital or health facility with a health post/ village clinic operated by MOH |
| CHAM/mission hospital | 374 | 3.56% | Linked to hospital or health facility operated by CHAM |
| CHAM/mission health center | 279 | 2.66% | Linked to hospital or health facility operated by CHAM |
| **Total included in comparison exercise** | **8,440** | **80.44%** |  |
| BLM | 693 | 6.61% | N/A |
| HSA | 452 | 4.31% | N/A |
| Private hospital / clinic | 328 | 3.13% | N/A |
| Mobile clinic | 173 | 1.65% | N/A |
| Shop | 170 | 1.62% | N/A |
| Private mobile clinic | 43 | 0.41% | N/A |
| CBDA / door to door | 31 | 0.30% | N/A |
| Friend / relative | 27 | 0.26% | N/A |
| CHAM/mission mobile clinic | 22 | 0.21% | N/A |
| Pharmacy | 19 | 0.18% | N/A |
| Private doctor | 11 | 0.10% | N/A |
| Private CBDA / door to door | 11 | 0.10% | N/A |
| Youth drop in center | 10 | 0.10% | N/A |
| CHAM/mission door to door | 6 | 0.06% | N/A |
| Church | 5 | 0.05% | N/A |
| Macro | 2 | 0.02% | N/A |
| Other | 29 | 0.28% | N/A |
| Other private medical | 1 | 0.01% | N/A |
| Don't know | 1 | 0.01% | N/A |
| Missing data | 18 | 0.17% | N/A |
| **Total ineligible for comparison exercise** | **2,052** | **19.56%** |  |
|  |  |  |  |
| **Grand Total** | **10,492** | **100%** |  |

*Legend: This table presents the number and percent of modern contraceptive users that are eligible for the study and those that are not, by last source of family planning method. It also demonstrates how a linkage was considered to be “appropriate”, when applicable*
