# Supplementary material for: Linking household surveys and facility assessments: a comparison of geospatial methods using nationally representative data from Malawi
Source: Popul Health Metr. 2020 Dec 10;18:30. doi: 10.1186/s12963-020-00242-z (PMC7731755; doi:10.1186/s12963-020-00242-z)
Supplement: Supplementary file 2 — Additional file 2:. Annex 2: GIS Methodologies. Legend: This document provides guidance on how to conduct the various linkages within a Geographic Information System. The authors hope that this will facilitate reproducibility and expand the use of these methods across settings. [file 12963_2020_242_MOESM2_ESM.docx]

## Annex 2: GIS Methodologies

*Legend: This document provides guidance on how to conduct the various linkages within a Geographic Information System. The authors hope that this will facilitate reproducibility and expand the use of these methods across settings.*

## Catchment Area Linking Methodology

**Creating Catchment Areas from a Comprehensive Free Health Service Registry**

Data Requirements:

- Complete registry of free health services
  - From highest level (hospitals) to lowest level (community outreach posts)
  - GPS coordinates for all points
  - Facility association for lower level community based points
- Operating GIS

Steps:

1. Add data of file containing GPS coordinates for free health services to GIS
2. Display XY coordinate data for all points
3. Export the coordinates to a new shapefile
4. Project coordinates to the working Projected Coordinate System (For Malawi: UTM 36S)
5. Create Thiessen Polygons for all of the points and save as a new shapefile
6. Dissolve the Thiessen Polygon shapefile based on the facility association value and save shapefile
7. Clip the output from step 6 to the study area in question

**Catchment Area Linkage**

Data Requirements

- Catchment area shapefile
- Population survey cluster point locations
- Operating GIS

Steps:

1. Add data files to project
2. Select “Spatial Join” tool
   1. Target: Cluster location
   2. Join: Catchment Areas
   3. Type of link: Join one to many
   4. Criteria: Target layer contains join layer

## Closest Facility Linking Methodology

Data Requirements:

- Facility point location
- Population survey cluster point locations
- Operating GIS

Steps:

1. Add files to project
2. Select “Spatial Join”
   1. Target: Clusters shapefile
   2. Join: Facility point location
   3. Type: One to one
   4. Criteria: Closest

## 5 Km Linking Methodology

Data Requirements:

- Facility point location
- Population survey cluster point locations
- Operating GIS

Steps:

1. Add files to project
2. Select “Spatial Join”
   1. Target: Clusters shapefile
   2. Join: Facility point location
   3. Type: One to many
   4. Criteria: Within a Distance (5 kilometers)

## Administrative Boundary Linking Methodology

Data Requirements:

- Clinic point location
- Population survey cluster point location
- Operating GIS

Steps:

1. Add data to project
2. Select by Attribute
   1. Enter all names of activity administrative areas and select
3. Export data to a new shapefile (Activity areas)
4. Select “Spatial Join”
   1. Target: Clusters shapefile
   2. Join: Activity areas
   3. Type: One to one
   4. Criteria: Within
